# Prognostic significance of the angiopoietin-2/angiopoietin-1 and angiopoietin-1/Tie-2 ratios for early sepsis in an emergency department

**DOI:** 10.1186/s13054-015-1075-6

**Published:** 2015-10-14

**Authors:** Yingying Fang, Chunsheng Li, Rui Shao, Han Yu, Qing Zhang, Lianxing Zhao

**Affiliations:** Emergency Department, Beijing Chao-yang Hospital, Capital Medical University, 8 Worker’s Stadium South Road, Beijing, Chao-yang District 100020 China

## Abstract

**Introduction:**

This study was performed to assess the early diagnostic, risk stratification, and prognostic value of the angiopoietin-2/angiopoietin-1 ratio (Ang-2/Ang-1) and angiopoietin-1/tyrosine kinase with immunoglobulin-like loop epidermal growth factor homology domain 2 ratio (Ang-1/Tie-2) and to compare these factors with procalcitonin (PCT) and the Mortality in Emergency Department Sepsis (MEDS) score in patients with early sepsis in the emergency department (ED).

**Methods:**

Consecutive patients with sepsis (n = 440) were enrolled in this study. They fulfilled the systemic inflammatory response syndrome (SIRS) criteria and were admitted to the ED of Beijing Chao-yang Hospital between August 2014 and February 2015. The control group consisted of 55 healthy blood donors. The patients were categorized into four groups: SIRS, sepsis, severe sepsis, and septic shock. Serum Ang-1, Ang-2, Tie-2, and PCT were measured, and the MEDS score was calculated upon ED arrival. The prognostic values of Ang-2/Ang-1, Ang-1/Tie-2, Ang-1, Ang-2, and Tie-2 were compared with the PCT and MEDS scores. All patients were followed for 28 days.

**Results:**

Upon admission, the median levels of the serum Ang-2 level and Ang-2/Ang-1 ratio increased and the serum Ang-1 levels and Ang-1/Tie-2 ratios decreased with the severity of sepsis. The areas under the receiver operating characteristic curves of the Ang-2/Ang-1 and Ang-1/Tie-2 ratios were greater than those of the Ang-1, Ang-2, and PCT levels and MEDS scores in the diagnosis and prediction of 28-day mortality due to sepsis. Ang-2/Ang-1 was significantly higher and Ang-1/Tie-2 was significantly lower in nonsurvivors than in survivors at the 28-day follow-up examination. Ang-2/Ang-1, Ang-1/Tie-2, and MEDS score were found to be independent predictors of 28-day mortality in patients with sepsis. The levels of serum Ang-1, Ang-2, and Tie-2 were positively correlated with each other. The ratios of Ang-2/Ang-1 and Ang-1/Tie-2 were positively and negatively correlated, respectively, with the MEDS score in every septic group.

**Conclusions:**

The Ang-2/Ang-1 and Ang-1/Tie-2 ratios are valuable for risk stratification in patients with sepsis and are associated with the poor clinical outcome of early sepsis in the ED.

## Introduction

Sepsis continues to be a very significant cause of mortality. Sepsis with multiple organ dysfunction syndrome (MODS) is the most catastrophic manifestation. In addition, patients who develop severe sepsis or septic shock have worse mortality than patients who do not develop sepsis [1, 2]. Effective management and resource allocation is difficult because of the inability to diagnose the severity and predict the high risk of sepsis. Although current biomarkers show great promise in indicating the severity of sepsis, the highly variable and nonspecific nature of the signs and symptoms of sepsis makes the prospect of single biomarker classification less valuable. Currently, it is of great significance to identify biomarkers and combine them with clinical scoring systems for risk stratification and evaluation of the prognosis of sepsis.

Among the complex mechanisms and heterogeneous nature of sepsis, widespread endothelial dysfunction plays an extremely important role in the severity of sepsis and sepsis-induced MODS [1, 3, 4]. The endothelium is a key target of sepsis-induced events, and the sepsis-activated vascular endothelium is responsible for the increase in luminal cell adhesion molecules, leukocyte recruitment, vasomotor tone alteration, microvascular thrombosis formation, and eventually diffuse capillary leakage [4, 5]. One of the most important mechanisms activating endothelial cells during sepsis is the endothelium-specific angiopoietin (Ang) tyrosine kinase with the immunoglobulin-like loop epidermal growth factor domain (Tie) ligand–receptor system, which has a potential correlation with endothelial injury severity. Ang-1, Ang-2, and Tie-2 play different roles in mediating vascular quiescence and inflammation. Ang-1 promotes vessel stability, suppresses inflammation, and promotes endothelial cell survival by activating the Tie-2 receptor complex [6–8], whereas Ang-2 destabilizes blood vessels, potentiates inflammation, and promotes proangiogenic effects, which result in vascular leakage and organ dysfunction by initially blocking the Tie-2 receptor [6, 9, 10]. The Tie-2 receptor is expressed and activated throughout the quiescent adult endothelium [11], where it promotes microvascular barrier function and anti-inflammation [12]. Investigators have investigated Ang-1 and Ang-2 in various studies as biomarkers of sepsis severity and mortality because of their roles in endothelial activation and their convenient measurement in the ED. Previous studies have shown that levels of Ang-1 and Ang-2 are clinically informative prognostic biomarkers of mortality in severe sepsis [13]. Ang-1 protects against organ dysfunction in animal models of sepsis [14], whereas Ang-2 is associated with sepsis severity and multiple organ dysfunction in sepsis in vitro [15] and in vivo [16]. However, in most of the previous studies, evidence for the use of the Ang-2/Ang-1 and Ang-1/Tie-2 ratios in predicting the severity and high mortality in patients with early sepsis has been lacking. Given these considerations, the aim of our present study was to investigate the correlation of serum the Ang-2/Ang-1 and Ang-1/Tie-2 ratios with risk stratification and prognostic evaluation of various degrees of early sepsis compared with PCT levels and MEDS scores.

## Material and methods

### Patients

This study was approved by the human research ethics committee of Beijing Chao-yang Hospital affiliated with the Capital Medical University (Beijing, China), and signed written informed consent forms were obtained from patients upon ED admission. The biological specimens and clinical data sets were obtained from a prospective observational study of patients who had a suspected infection with two or more criteria of systemic inflammatory response syndrome (SIRS) and who were enrolled in the ED of Beijing Chao-yang Hospital, which had an average of 260,000 visits annually during the period between August 2014 and January 2015. The patient records were anonymous and deidentified before analysis. The exclusion criteria were (1) age <18 years old or >80 years old; (2) pregnancy or breastfeeding; (3) neutropenia, defined as <1000 neutrophils/mm^3^; (4) HIV infection; (5) hypertension or constantly taking angiotensin-converting enzyme inhibitors, angiotensin II receptor antagonists, or renin inhibitors; (6) chronic intake of corticosteroids, defined as any daily oral intake of 1 mg/kg or more of equivalent prednisone for more than 1 month; (7) recent use of doses of unfractionated or low-molecular-weight heparin; and (8) lack of informed consent by the patients or their relatives.

A total of 440 patients who were followed for 28 days or until death were categorized on the basis of the 2001 Society of Critical Care Medicine/European Society of Intensive Care Medicine/American College of Chest Physicians/American Thoracic Society/Surgical Infection Society International Sepsis Definitions Conference and Surviving Sepsis Campaign guidelines [17, 18] into the following subgroups: SIRS (n = 107), sepsis (n = 176), severe sepsis (n = 84), and septic shock (n = 73). The control group included 55 healthy blood donors with no history or clinical evidence of acute or chronic disease. They were similar in age and sex to the experimental groups.

### Sample collection and measurements

Most of the blood samples were obtained from blood drawn for routine diagnostic procedures, and no additional blood was obtained from the patients. Informed consent was provided by the patients or their first-degree relatives and by healthy control subjects before blood collection. Within 15 min of arrival in the ED, a 5-ml sample of blood was collected from patients and healthy donors in tubes containing ethylenediaminetetraacetic acid. The samples were immediately transferred to the local central laboratory. The serum was stored at −80 °C until further measurement.

Samples were analyzed by researchers who were blinded to all patient data. Serum Ang-1, Ang-2, and Tie-2 were measured in available samples upon admission using an enzyme-linked immunosorbent assay (Abcam, Cambridge, MA, USA). PCT levels were measured using an enzyme-linked fluorescence immunoassay with a miniVIDAS immunoassay analyzer (bioMérieux, Durham, NC, USA). All standards, controls, and test samples were assayed in duplicate. All measurements were performed according to the manufacturers’ instructions.

### Calculation of the MEDS score and APACHE II score

The MEDS score was calculated by summing the points of nine variables: terminal illness, tachypnea or hypoxia, septic shock, low platelet count, age >65 years, lower respiratory tract infection, nursing home resident, and altered mental status [19]. The Acute Physiology and Chronic Health Evaluation (APACHE) II score consisted of measurements of the body temperature, mean arterial pressure, heart rate, respiratory rate, oxygenation, arterial pH, sodium, potassium, creatinine, hematocrit, white blood cell count, Glasgow Coma Scale score, age, chronic diseases, and surgical status, with a total possible score between 0 and 71 [20].

### Statistical analyses

These results were presented as the mean standard deviation or median range (interquartile range). Nonnormal distribution of continuous variables was tested using the Kolmogorov-Smirnov test and Mann–Whitney *U* test. In patients, differences between survivors and nonsurvivors were also compared using a nonparametric two-sided Mann–Whitney *U* test. Comparisons between groups are illustrated with box plot graphics, where the dotted line indicates the median, the box represents the 25th–75th percentiles, the horizontal lines show the minimum and maximum values of the calculated nonoutlier values, and the open circles indicate outlier values. To determine independent predictors of 28-day mortality, logistic regression analysis was performed while adjusting for age, sex, type of infection, and infection sites. A receiver operating characteristic (ROC) curve was used to assess the accuracy of the variables in prediction of 28-day mortality. In addition, the comparisons of areas under the ROC curves (AUCs) were analyzed using MedCalc version 11.6 software (MedCalc Software, Ostend, Belgium). Furthermore, the optimal cutoff value for Ang-2/Ang-1 and Ang-1/Tie-2 ratios to differentiate between survivors and nonsurvivors was determined empirically from the ROC curve using the Youden index [21]. Two-sided *p* values of 0.05 were considered statistically significant. Confidence intervals (CIs) were reported at 95 %. Kaplan–Meier survival curves were constructed for patients to examine the prognostic value of the Ang-2/Ang-1 and Ang-1/Tie-2 ratios according to their levels at ED admission. The log-rank test was used to compare survival rates between groups. Finally, Spearman’s rank correlation coefficient was calculated to describe correlations between Ang-1, Ang-2, and Tie-2 levels, as well as Ang-2/Ang-1 ratio, Ang-1/Tie-2 ratio, and MEDS score. All of the data were analyzed using IBM SPSS 19.0 software (IBM, Armonk, NY, USA).

## Results

### Patient characteristics

**Fig. 1:**
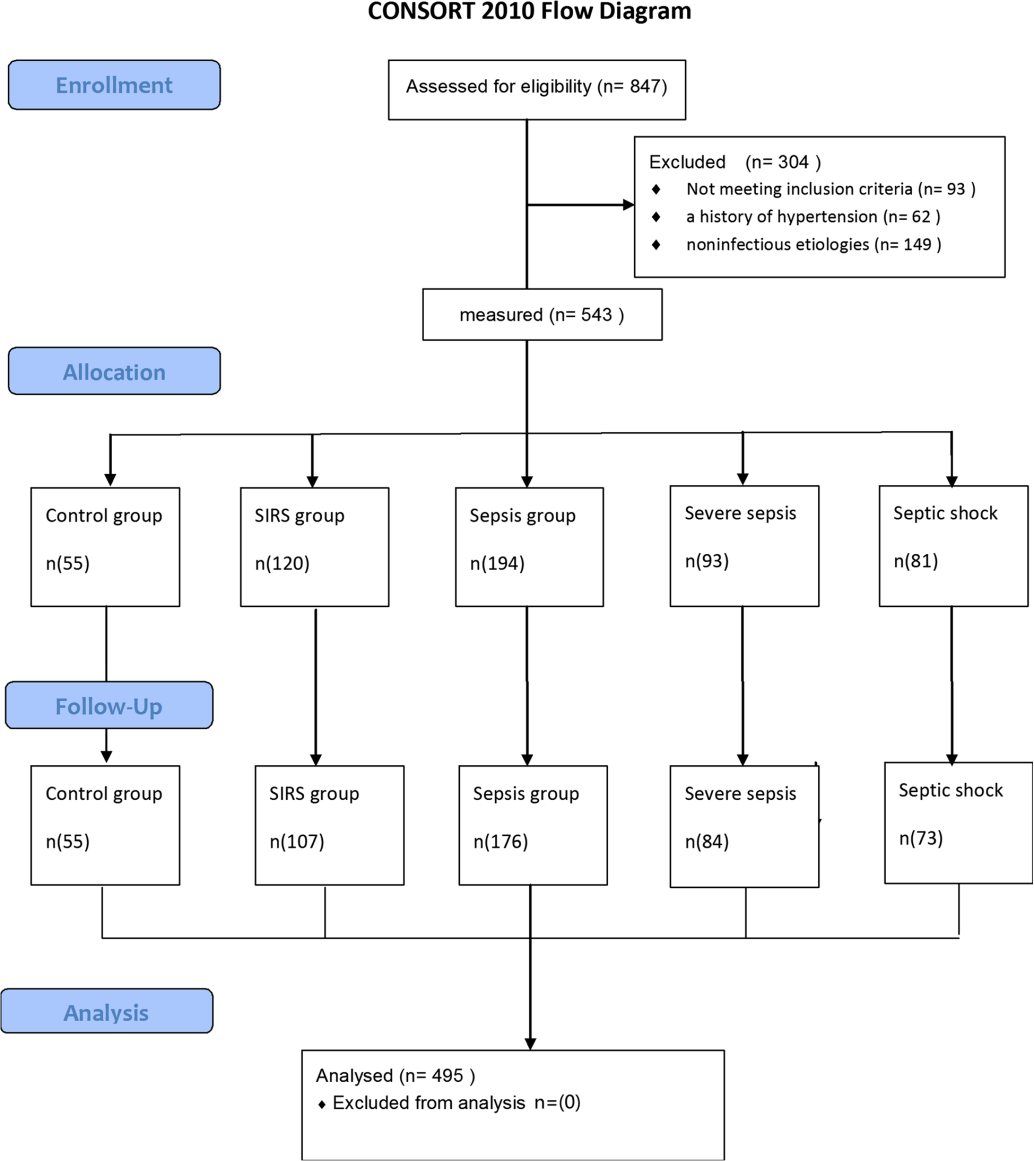
CONSORT flow diagram of the study. *SIRS* systemic inflammatory response syndrome

A total of 440 patients and 55 healthy controls were enrolled in the study and the recruitment of patients into the study is shown in Fig. 1. There were no significant differences in age, sex, heart rate (HR), ratio of partial pressure arterial oxygen to fraction of inspired oxygen, infections between the five groups (healthy controls, SIRS, sepsis, severe sepsis, and septic shock). There were significant differences between groups in the APACHE II and MEDS scores (*p* < 0.0001). The basic characteristics, mediators and infections of the enrolled subjects are listed in Tables 1 and 2.

**Table 1:** Basic characteristics of the study population

Characteristics	Control	SIRS	Sepsis	Severe sepsis	Septic shock	*p* value
Number of subjects	55	107	176	84	73	
Demographic data						
Age (yr)	61 (54–71)	68 (56–76)	71 (59–78)	74 (61–78)	75 (65–78)	0.202
Male (%)	54.5	52.6	61.0	60.3	58.1	0.831
Physiological data						
MAP (mmHg), mean ± SD	100 ± 19	110 ± 21	90 ± 15	89 ± 13	64 ± 14	0.042
HR (beats/min), mean ± SD	79 ± 22	103 ± 21	99 ± 15	105 ± 23	105 ± 23	0.339
PaO_2_/FIO_2_ ratio	306 (258–379)	300 (255–369)	293 (245–363)	235 (130–295)	213 (103–265)	0.098
APACHE II score	0	6.00 (3.00–8.00)	7.00 (6.07–9.00)	15.5 (10.0–22.0)	21.0 (16.1–29.0)	<0.0001
MEDS score	0	5.00 (3.00–6.00)	7.00 (6.00–8.00)	19.0 (18.0–22.0)	25.0 (24.0–26.0)	<0.0001

*Abbreviations: SD* standard deviation, *SIRS* systemic inflammatory response syndrome, *APACHE* Acute Physiology and Chronic Health Evaluation, *MEDS* Mortality in Emergency Department Sepsis, *MAP* mean arterial pressure, *HR* heart rate, *PaO*_*2*_ */FiO*_*2*_ ratio of partial pressure arterial oxygen to fraction of inspired oxygen

**Table 2:** Mediators and infections of the study population

	Control	SIRS	Sepsis	Severe sepsis	Septic shock	*p* value
Number of subjects	55	107	176	84	73	
Mediator levels						
PCT (ng/ml)	0.11 (0.09–0.13)	0.64 (0.56–0.71)	1.20 (1.09–6.67)	7.97 (3.06–12.3)	13.2 (1.21–33.0)	<0.0001
Ang-1 (ng/ml)	9.54 (7.56–10.89)	9.11 (5.67–11.2)	7.57 (6.78–8.75)	5.32 (4.56–6.24)	3.06 (0.99–7.45)	<0.0001
Ang-2 (ng/ml)	3.41 (1.45–6.23)	5.42 (5.18–5.91)	13.0 (12.0–6.24)	18.7 (12.1–19.4)	15.7 (11.4–27.8)	<0.0001
Tie-2 (ng/ml)	107.9 (92.1–129.3)	113.0 (99.8–189.9)	174.7 (134.2–220.4)	272.1 (176.5–358.4)	311.9 (149.9–415.2)	<0.0001
Ang-2/Ang-1 ratio	0.49 (0.16–0.61)	0.73 (0.51–0.91)	1.73 (1.37–2.00)	3.42 (1.93–4.15)	5.14 (1.50–18.95)	<0.0001
Ang-1/Tie-2 ratio (×10^3^)	82.4 (68.3–98.0)	63.0 (40.6–84.4)	43.1 (34.9–62.2)	17.0 (11.5–28.8)	8.7 (1.8–41)	<0.0001
Infection data (n)						
Respiratory (lung)		AECOPD (77)	Pneumonia (121)	Pneumonia (60)	Pneumonia (50)	0.564
		Asthma (9)				
		PE (2)				
Intra-abdominal sites		Pancreatitis (7)	IAI (24)	IAI (14)	IAI (15)	0.558
Cerebral		Stroke (8)	Meningitis (15)	Meningitis (5)	Meningitis (6)	0.695
Urinary			Pyelonephritis (10)	Pyelonephritis (5)	Pyelonephritis (2)	0.754
Other		DKA (4)	Skin/soft tissue infection (6)			
28-day mortality, n (%)	0	15 (14.0)	32 (18.2)	36 (42.9)	45 (61.6)	<0.0001
Missing data, n (%)	0	13 (10.8)	18 (9.3)	9 (9.7)	8 (9.9)	0.770

*Abbreviations: SIRS* systemic inflammatory response syndrome, *PCT* procalcitonin, *Ang* angiopoietin, *Tie-2* tyrosine kinase with immunoglobulin-like loop epidermal growth factor domain 2, *AECOPD* acute exacerbations of chronic obstructive pulmonary disease, *PE* pulmonary embolism, *RI* respiratory infection, *IAI* intraabdominal infection, *DKA* diabetic ketoacidosis

### Comparison of median Ang-1, Ang-2, and Tie-2 levels, Ang-2/Ang-1 ratio, Ang-1/Tie-2 ratio, and MEDS score

**Fig. 2:**
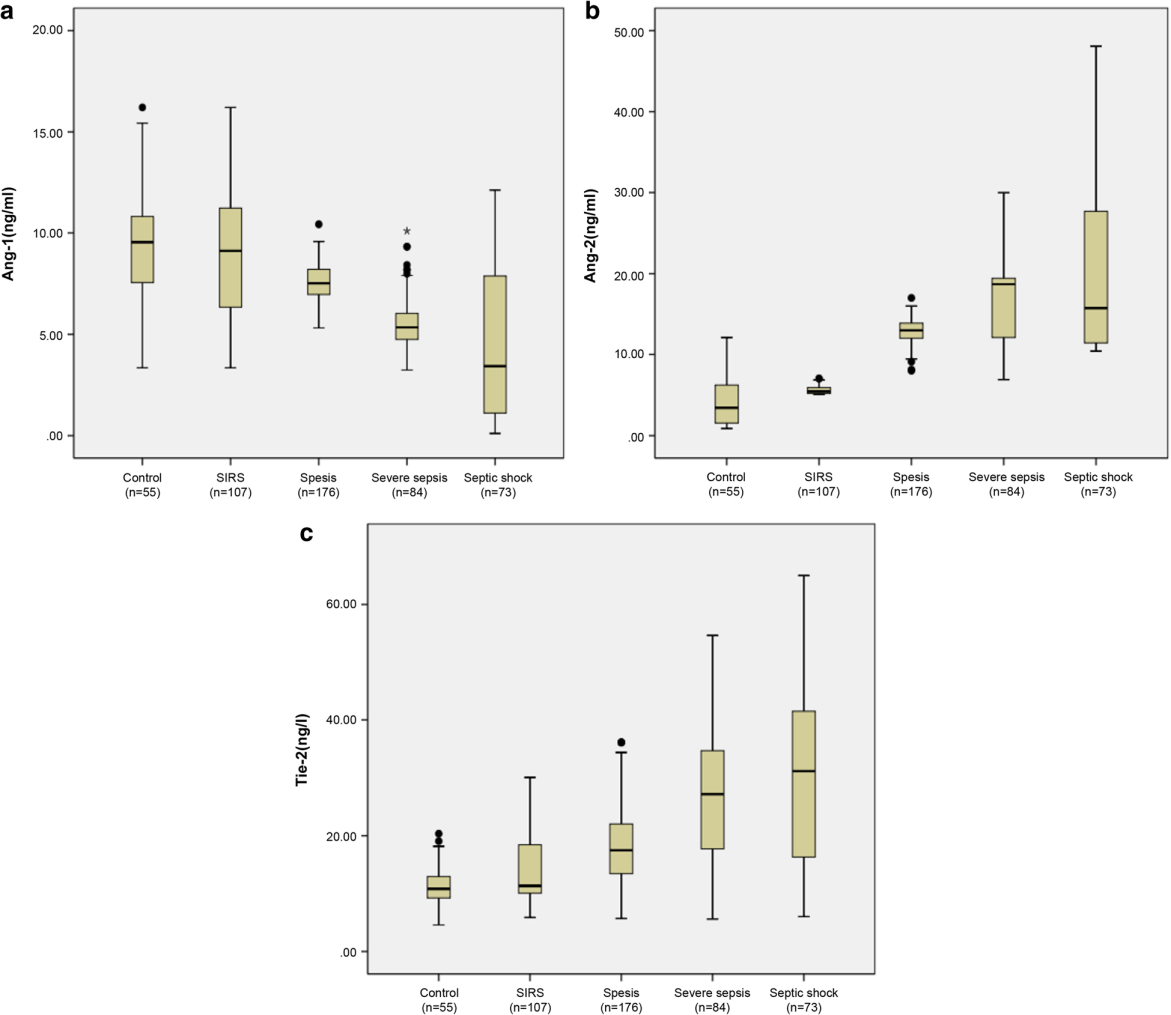
The serum levels of Ang-1 (**a**), Ang-2 (**b**), and Tie-2 (**c**) in healthy control individuals and in patients with systemic inflammatory response syndrome, sepsis, severe sepsis, and septic shock upon emergency department admission. *Ang* angiopoietin, *SIRS* systemic inflammatory response syndrome, *Tie-2* tyrosine kinase with immunoglobulin-like loop epidermal growth factor homology domain 2

Table 2 shows, for each study group, the median Ang-1, Ang-2, and Tie-2 levels; Ang-2/Ang-1, and Ang-1/Tie-2 ratios; the PCT level; and the MEDS score. Figure 2 displays the Ang-1, Ang-2, and Tie-2 levels in each group. Serum Ang-1, Ang-2, Tie-2, and PCT and the MEDS score at ED admission were significantly different among the groups. Compared with the healthy control group, the Ang-2, Tie-2, PCT level, and MEDS score were significantly higher, and Ang-1 level was lower, in patients with SIRS, sepsis, severe sepsis, and septic shock (*p* < 0.0001). Interestingly, the Ang-2/Ang-1 and Ang-1/Tie-2 ratios were markedly different in sepsis, severe sepsis, and septic shock compared with the control group (*p* < 0.0001).

### Ang-2/Ang-1 and Ang-1/Tie-2 ratios in survivors and nonsurvivors

**Fig. 3:**
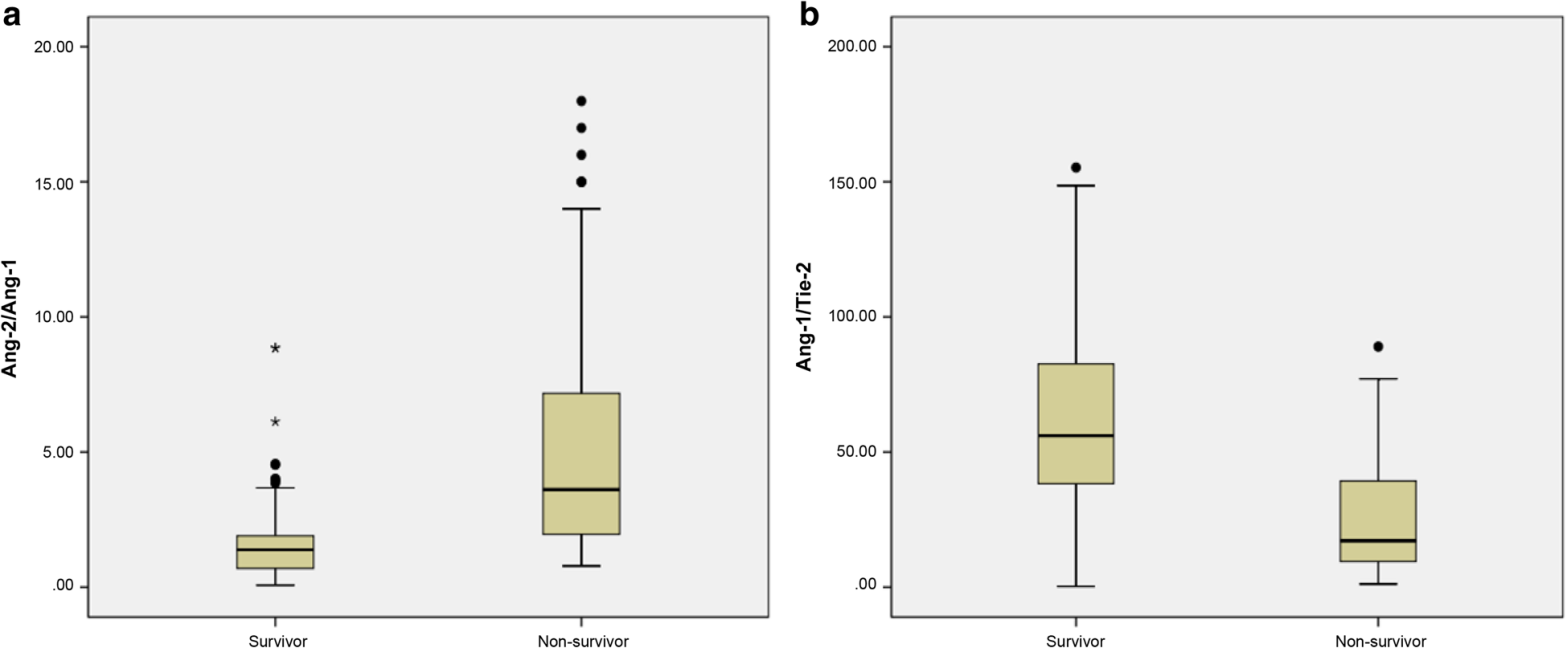
Ang-2/Ang-1 (**a**) and Ang-1/Tie-2 (**b**) ratios at admission in survivor and nonsurvivor groups of patients with sepsis at 28-day follow-up. *Lines* denote he median values, *boxes* represent 25th–75th percentiles, and *whiskers* indicate the range. *Ang* angiopoietin, *Tie-2* tyrosine kinase with immunoglobulin-like loop epidermal growth factor homology domain 2

The Ang-2/Ang-1 ratio was higher in survivors than in nonsurvivors, whereas the Ang-1/Tie-2 ratio was lower in survivors than in nonsurvivors (*p* < 0.0001) (Fig. 3).

### Value of the Ang-2/Ang-1 and Ang-1/Tie-2 ratios and MEDS scores for predicting 28-day mortality

The AUC of Ang-2/Ang-1 ratio for predicting 28-day mortality in patients with sepsis was 0.845 higher than PCT (0.732) and Ang-1 and Ang-2 levels (0.778 and 0.794, respectively) (all *p* < 0.05). The AUC of Ang-1/Tie-2 ratio for predicting 28-day mortality in patients with sepsis was 0.808 higher than PCT (0.732) (*p* < 0.05). The AUC of Ang-2/Ang-1 ratio for predicting 28-day mortality was 0.845, higher than that of MEDS score (0.826), but was not statistically significant (*p* >0.05). The AUC of Ang-2/Ang-1 ratio in combination with the MEDS score was 0.857, which was statistically significant compared with MEDS score (0.826) (*p* < 0.05). The AUC of Ang-1/Tie-2 ratio in combination with MEDS score was higher than MEDS score alone (0.844 vs. 0.826; *p* < 0.05).

**Fig. 4:**
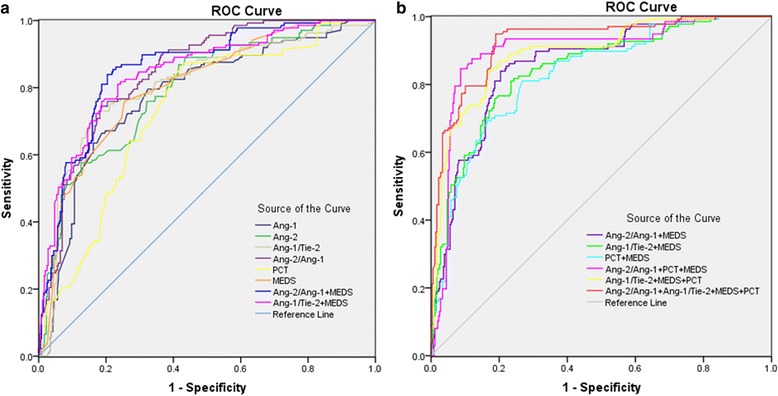
ROC curves for predicting 28-day mortality in patients with sepsis. **a** AUCs for predicting 28-days mortality: Ang-2/Ang-1 ratio in combination with MEDS score = 0.857 (*blue line*), Ang-1/Tie-2 ratio in combination with MEDS score = 0.844 (*pink line*). **b** Similarly, AUCs for predicting 28-days mortality: Ang-2/Ang-1 ratio in combination with MEDS = 0.857 (*purple line*), Ang-1/Tie-2 ratio in combination with MEDS score = 0.844 (*green line*), PCT in combination with MEDS score = 0.829 (*blue line*), a combination of Ang-2/Ang-1 ratio, MEDS score, and PCT = 0.900 (*pink line*), a combination of Ang-1/Tie-1 ratio, MEDS score, and PCT = 0.894 (*yellow line*), a combination of Ang-2/Ang-1, Ang-1/Tie-1 ratios, MEDS score, and PCT = 0.925 (*red line*). *Ang* angiopoietin, *AUC* area under the receiver operating characteristic curve, *MEDS* Mortality in Emergency Department Sepsis, *PCT* procalcitonin, *ROC* receiver operating characteristic, *Tie-2* tyrosine kinase with immunoglobulin-like loop epidermal growth factor homology domain 2

The AUC for a combination of Ang-2/Ang-1 ratio, MEDS score, and PCT was significantly higher than that for PCT combined with MEDS score (0.900 vs. 0.829; *p* < 0.01). The AUC for a combination of Ang-1/Tie-2 ratio, MEDS score, and PCT was also significantly higher than that of PCT combined with MEDS score (0.894 vs. 0.829; *p* < 0.05). Among the prognostic factors studied, the best rate for prediction of 28-day mortality was for a combination of Ang-2/Ang-1 and Ang-1/Tie-2 ratios, MEDS score, and PCT, which was significantly higher than for each parameter alone (*p* < 0.01). All of the data were analyzed using MedCalc version 11.6 software. The ROC curves of these biomarkers and MEDS scores for predicting 28-day mortality among the five groups are shown in Fig. 4.

Using an Ang-2/Ang-1 ratio cutoff value of 1.94 for predicting 28-day mortality in patients with sepsis, the sensitivity was 79.7 %, the specificity was 81.3 %, the positive predictive value (PPV) was 78.4 %, the negative predictive value (NPV) was 82.5 %, the positive likelihood ratio (LR+) was 3.98, and the negative likelihood ratio (LR−) was 0.31. Using an Ang-1/Tie-2 ratio cutoff value of 30.0 for predicting 28-day mortality in patients with sepsis, the sensitivity was 73.0 %, the specificity was 82.4 %, the PPV was 79.4 %, the NPV was 77.9 %, the LR+ was 4.15, and the LR– was 0.33. The detailed results are presented in Tables 3 and 4.

**Table 3:** AUCs for predicting 28-day mortality in patients with early sepsis

	Variable	AUC	Standard error	*p* value	95 % CI	
					Lower limit	Upper limit
28-day mortality	Ang-1	0.778	0.024	0	0.732	0.824
	Ang-2	0.794	0.018	0	0.750	0.837
	Tie-2	0.803	0.024	0	0.756	0.849
	Ang-2/Ang-1	0.845	0.018	0	0.810	0.880
	Ang-1/Tie-2	0.808	0.023	0	0.763	0.853
	PCT	0.732	0.024	0	0.685	0.780
	MEDS score	0.826	0.021	0	0.785	0.866
	Ang-2/Ang-1+MEDS score	0.857	0.018	0	0.821	0.893
	Ang-1/Tie-2+MEDS score	0.844	0.020	0	0.805	0.883
	PCT+MEDS score	0.829	0.020	0	0.790	0.869
	Ang-2/Ang-1+PCT+MEDS score	0.900	0.017	0	0.866	0.934
	Ang-1/Tie-2+PCT+MEDS score	0.894	0.016	0	0.862	0.926
	Ang-2/Ang-1+Ang-1/Tie-2+PCT+MEDS score	0.925	0.013	0	0.898	0.951

*Abbreviations: Ang* angiopoietin, *AUC* area under the receiver operating characteristic curve, *CI* confidence interval, *MEDS* Mortality in Emergency Department Sepsis, *PCT* procalcitonin, *Tie-2* tyrosine kinase with immunoglobulin-like loop epidermal growth factor homology domain 2

**Table 4:** Performance of multivariable models for predicting 28-day mortality in patients with early sepsis

	Variable	Cutoff	Sensitivity (%)	Specificity (%)	PPV (%)	NPV (%)	LR+	LR−
28-day mortality	Ang-2/Ang-1	1.94	79.7	81.3	78.4	82.5	3.98	0.31
	Ang-1/Tie-2 (×10^3^)	30.0	73.0	82.4	79.4	77.9	4.15	0.33
	PCT	5.27	62.3	84.9	60.3	85.4	2.03	0.22
	MEDS score	14.5	70.6	76.3	56.2	90.7	3.35	0.27

*Abbreviations: Ang* angiopoietin, *LR−* negative likelihood ratio, *LR+* positive likelihood ratio, *MEDS* Mortality in Emergency Department Sepsis, *NPV* negative predictive value, *PCT* procalcitonin, *PPV* positive predictive value, *Tie-2* tyrosine kinase with immunoglobulin-like loop epidermal growth factor homology domain 2

### Ang-2/Ang-1 ratio and MEDS score as independent predictors of 28-day mortality

Age, sex, Ang-1, Ang-2, and Tie-2 levels, Ang-2/Ang-1 ratio, Ang-1/Tie-2 ratio, PCT, and MEDS score were included in a multivariate logistic regression model to determine the independent predictors of 28-day mortality. Ang-2/Ang-1 (β = −0.013, odds ratio [OR] = 0.987, *p* < 0.0001), Ang-1/Tie-2 (β = −6.553, OR = 0.011, *p* = 0.004), and MEDS score (β = 0.167, OR = 1.182, *p* < 0.0001) were found to be independent predictors of 28-day mortality in patients with sepsis (Table 5).

**Table 5:** Independent predictors of 28-day mortality in patients with early sepsis

	Variable	β	Standard error	Wald statistic	Degrees of freedom	*p* value	Odds ratio	95 % CI	
								Lower limit	Upper limit
28-day mortality	Ang-1	−0.063	0.081	0.605	1	0.157	0.939	0.802	1.100
	Ang-2	0.067	0.022	8.996	1	0.003	1.069	1.023	1.117
	Tie-2	0.002	0.002	0.807	1	0.269	1.002	0.998	1.072
	Ang-2/Ang-1	−0.013	0.043	0.094	1	0.026	0.987	0.909	1.072
	Ang-1/Tie-2	−6.553	7.793	0.707	1	0.024	0.011	0.009	0.013
	PCT	−0.006	0.019	0.101	1	0.325	0.994	0.958	1.032
	MEDS score	0.167	0.028	35.637	1	0.009	1.182	1.119	1.249
	Constant	3.901	1.585	6.058	1	0.714	0.432		

*Abbreviations: Ang* angiopoietin; *CI* confidence interval, *MEDS* Mortality in Emergency Department Sepsis, *PCT* procalcitonin, *Tie-2* tyrosine kinase with immunoglobulin-like loop epidermal growth factor homology domain 2

### Survival

**Fig. 5:**
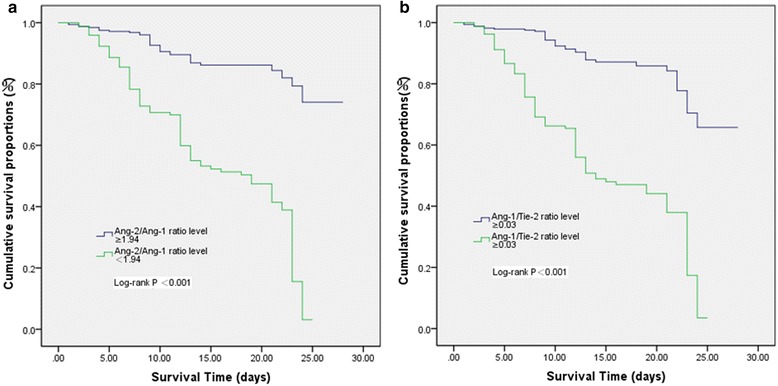
**a** Kaplan–Meier survival curves show that patients with sepsis with Ang-2/Ang-1 ratios higher than 1.94 had a lower probability of survival at 28 days (log-rank = 94.42; *p* < 0.0001) compared with patients with lower levels. **b** Similarly, patients with sepsis with Ang-1/Tie-2 ratios lower than 0.03 had a lower probability of survival at 28 days (log-rank = 100.48; *p* < 0.0001) than patients with lower levels. *Ang* angiopoietin, *Tie-2* tyrosine kinase with immunoglobulin-like loop epidermal growth factor homology domain 2

Using cutoff values determined by ROC curves, Kaplan–Meier survival curves were established. Figure 5a illustrates the Kaplan–Meier curves of 28-day survival stratified by the admission levels of the Ang-2/Ang-1 ratio above and below 1.94. The log-rank test confirmed the statistical significance of Ang-2/Ang-1 (log-rank = 94.42; *p* < 0.0001). Similarly, Kaplan–Meier survival analysis also showed that patients with sepsis with a serum Ang-1/Tie-2 ratio lower than 30.0 had a higher probability of survival at 28 days, as shown in Fig. 5b (log-rank = 100.48; *p* < 0.0001).

### Correlation of serum levels of Ang-1 and Ang-2 with serum Tie-2 levels and Ang-2/Ang-1 ratio, Ang-1/Tie-2 ratio with MEDS score

Spearman’s correlation analysis of Ang-1 and Ang-2 with Tie-2 showed correlation coefficients of 0.574, −0.613, and 0.637, respectively (all *p* < 0.0001). The correlation coefficients between Ang-2/Ang-1, Ang-1/Tie-2, and MEDS score were 0.527 and −0.555, which suggested a positive and negative correlation, respectively (all *p* < 0.0001).

## Discussion

It is important to identify useful biomarkers to improve early diagnosis and risk stratification, particularly in the early stages of sepsis. In our study, patients with sepsis presented with significantly decreased serum Ang-1 levels and Ang-1/Tie-2 ratios and markedly elevated serum Ang-2 and Tie-2 levels and Ang-2/Ang-1 ratios measured at admission [13, 22, 23]. The Ang-2/Ang-1 and Ang-1/Tie-2 ratios are considered to be valuable biomarkers in rapid risk stratification and prognosis for 28-day mortality in patients with sepsis in the ED.

Researchers in two other studies to date have reported Ang-2/Ang-1 ratios in patients with sepsis, and the evidence of similar Ang-1/Tie-2 ratios has been lacking. In the first study, Ricciuto et al. found that the admission levels of Ang-2/Ang-1 ratio at baseline were not robust markers for 28-day mortality in patients with severe sepsis [23]. In the second study, Wang et al. showed that the Ang-2/Ang-1 ratio was not a promising marker for severity or outcomes in pediatric patients with severe sepsis and less effective than using Ang-1 and Ang-2 alone [24]. The discrepancy between our observations and those of Ricciuto et al. may relate to the fact that Ang-2/Ang-1 ratio was measured in plasma in their study, not in serum as in ours. Also, our study population was consecutively collected and comprised a large number of subjects (n = 495), whereas only 70 patients with severe sepsis were enrolled in their patient cohort. Their study’s small sample size might have been responsible for an underpowered or erroneous multivariable analysis lacking differences between the groups. In addition, our study population contained various degrees of early sepsis and was well divided into control (n = 55), SIRS (n = 107), sepsis (n = 176), severe sepsis (n = 84), and septic shock (n = 73), whereas their study cohort included merely patients with severe sepsis divided into survivors (n = 39) and nonsurvivors (n = 31) at 28 days and did not include healthy individuals. The discrepancies between our results and those of Wang et al. may be the greatly different pathophysiology between critically ill pediatric patients and adults, along with the complex and heterogeneous nature of sepsis. Moreover, the relatively small number of pediatric patients with severe sepsis sampled (n = 45) might have been another reason resulting in a lack of differences between the groups.

In the developed vasculature, Ang-1 protects against vascular leakage, whereas Ang-2 promotes increased vascular permeability. Ang-1 and Ang-2 appear to act as an agonist–antagonist pair mediating capillary endothelium leakage (i.e., Ang-2/Ang-1). The higher the Ang-1/Ang-2 ratio is, the more severe the capillary endothelial damage. In addition, Ang-1 has been proposed to be necessary for the maintenance of vascular barrier function via its interaction with Tie-2 in blood vessels. As a default pathway, constitutive Ang-1–Tie-2 interactions promote endothelial cell migration, proliferation, survival, and differentiation and regulate endothelial barrier integrity and stability [25, 26]. A lower Ang-1/Tie-2 ratio indicates a stronger defense against capillary endothelial damage. Taken together, the increase in the Ang-2/Ang-1 ratio and the decrease in the Ang-1/Tie-2 ratio demonstrate that destruction of the capillary endothelium is greater than the stability in the deterioration of sepsis. Thus, high admission serum Ang-2/Ang-1 ratio and low admission serum Ang-1/Tie-2 ratio identify the severity of sepsis in patients at the onset of early sepsis in the ED.

The Ang-2/Ang-1 and Ang-1/Tie-2 ratios were superior to the absolute Ang-1 and Ang-2 levels for prognostic purposes in critical care [27, 28] and had significant diagnostic and prognostic value for 28-day mortality. Our results show that an initial relative deficiency of Ang-1 level associated with a sharp increase in Ang-2 level is associated with risk stratification and clinically poorer outcome in patients with sepsis. The AUC of Ang-2/Ang-1 ratio for predicting 28-day mortality in patients with sepsis was higher than the AUCs for Ang-1 and Ang-2 levels. On the one hand, Chong and colleagues [29] recently determined that a “relative deficiency” of Ang-1 levels and a “relative adequacy” of Ang-2 levels most likely indicate the degree of capillary endothelial injury in sepsis and in patients with sepsis with high mortality arising from a more severe degree of injury to the capillary endothelium, resulting in relative changes of Ang-1 and Ang-2. Thus, the ratio between these two contrasting angiogenesis factors may be capable of determining the fate of capillary endothelial cells, and Ang-2/Ang-1 ratio is likely to be a strong biomarker of sepsis mortality. On the other hand, in in vivo and vitro studies, Mofarrahi and colleagues found that lipopolysaccharide administration downregulates Ang-1 expression and functionally inhibits the Ang-1/Tie-2 receptor pathway, resulting in an imbalance of endothelial barrier integrity and stability and subsequent organ dysfunction. This is the main reason for the use of the Ang-1/Tie-2 ratio as a prognostic biomarker of sepsis [26].

Currently, PCT is the most studied and promising sepsis biomarker for diagnosis, risk stratification, evaluation of prognosis, and therapy monitoring [30]. Previous studies of the utility of PCT have been focused mainly on their diagnostic value for bacterial infection and are not a specific indicator of all types of infection in sepsis [31]. A substantial number of studies have challenged the prognostic role of PCT [32]. In addition, the MEDS score is more suitable than the other severity scoring systems for predicting mortality among patients with sepsis in the ED, despite the controversial value of clinical application [33]. In our study, the PCT level and MEDS score were chosen as a variable to compare with the Ang-2/Ang-1 and Ang-1/Tie-2 ratios.

Because there are advantages and disadvantages of biomarkers and scoring systems in the prognosis of sepsis, the combination between biomarkers with severity scoring systems can overcome their limitations and improve the accuracy of prognosis. In our study, the combination of the Ang-2/Ang-1 and Ang-1/Tie-2 ratios with the MEDS score was more effective than that of PCT or the MEDS score alone in predicting 28-day mortality. PCT was used merely to support the diagnosis of bacterial infection, but it exhibited no discriminative power for the prediction of mortality in early sepsis. Although the MEDS score is regarded as an easy and reliable method for predicting mortality in patients with a suspected infection, the discriminative ability of the combination of PCT and the MEDS score in the prognostic evaluation of sepsis is still limited. However, in a more comprehensive aspect, the combination of the Ang-2/Ang-1 and Ang-1/Tie-2 ratios with the MEDS score indicates the degree of general capillary endothelial damage and pathological condition of patients with sepsis and reflects the pathophysiological mechanism of widespread capillary endothelium and the risk of short-term mortality of patients in early sepsis. Taken together, the Ang-2/Ang-1 and Ang-1/Tie-2 ratios enhanced the accuracy of PCT and the MEDS score in prognostic evaluation.

In addition, there were significant close correlations between the Ang-1, Ang-2, and Tie-2 levels [6] and between the Ang-2/Ang-1 or Ang-1/Tie-2 ratios and the MEDS score. Similar associations between the Ang-2/Ang-1 or Ang-1/Tie-2 ratios and the MEDS score in critically ill patients have not been reported previously. The survival curves clearly illustrated the optimized information provided by the use of the Ang-2/Ang-1 or Ang-1/Tie-2 ratios. Both markers above or below their respective cutoff value indicate a high risk of death.

### Limitations

Some limitations of this study merit consideration. First, convenience sampling or the subjective inclusion criterion of “suspicion of infection” may have introduced bias into the clinical portion of this work. Second, the patient information on pathogenic evidence and treatment preferences was not considered in the initial study. Third, comparisons of the Ang-1, Ang-2, and Tie-2 levels before and after treatment were not performed during the patients’ stay in the ED. Fourth, the complicated underlying diseases might have had an effect on the results.

## Conclusions

Serum Ang-2/Ang-1 and Ang-1/Tie-2 ratios are independent predictors of 28-day mortality in patients with sepsis in the ED. The serum Ang-2/Ang-1 and Ang-1/Tie-2 ratios are associated with risk stratification and prognostic evaluation of sepsis and are more valuable predictors of 28-day mortality than PCT and MEDS score.

## Key messages

- The serum Ang-2/Ang-1 and Ang-1/Tie-2 ratios were good biomarkers for reflecting the severity of sepsis.
- The serum Ang-2/Ang-1 and Ang-1/Tie-2 ratios were superior to PCT and MEDS score for predicting 28-day mortality in patients with sepsis in the ED.
- The serum Ang-2/Ang-1 and Ang-1/Tie-2 ratios and MEDS score were all independent predictors of 28-day mortality in patients with sepsis.
- The serum Ang-2/Ang-1 or Ang-1/Tie-2 ratios, in combination with PCT and MEDS score, enhanced the predictive accuracy of 28-day mortality in patients with sepsis.

## Abbreviations

AECOPD: acute exacerbations of chronic obstructive pulmonary disease
Ang: angiopoietin
APACHE: Acute Physiology and Chronic Health Evaluation
AUC: area under the receiver operating characteristic curve
CI: confidence interval
CONSORT: Consolidated Standards of Reporting Trials
DKA: diabetic ketoacidosis
ED: emergency department
HR: heart rate
IAI: intraabdominal infection
LR−: negative likelihood ratio
LR+: positive likelihood ratio
MAP: mean arterial pressure
MEDS: Mortality in Emergency Department Sepsis
MODS: multiple organ dysfunction syndrome
NPV: negative predictive value
OR: odds ratio
PaO_2_/FiO_2_: ratio of partial pressure arterial oxygen to fraction of inspired oxygen
PCT: procalcitonin
PE: pulmonary embolism
PPV: positive predictive value
RI: respiratory infection
ROC: receiver operating characteristic
SD: standard deviation
SIRS: systemic inflammatory response syndrome
Tie-2: tyrosine kinase with immunoglobulin-like loop epidermal growth factor homology domain 2
